# Neurological Manifestations in a Cohort of Egyptian Patients with COVID-19: A Prospective, Multicenter, Observational Study

**DOI:** 10.3390/brainsci12010074

**Published:** 2022-01-01

**Authors:** Doaa A. Mekkawy, Sherif Hamdy, Maged Abdel-Naseer, Hatem S. Shehata, Ahmed Al Halfawy, Nevin M. Shalaby, Ghaydaa A. Shehata, Anwar M. Ali, Alaa Elmazny, Sandra M. Ahmed, Jumana H. Ismail, Aml Ibraheim, Hoda M. Abdel-Hamid, Rehab Magdy, Younan Kabara Ayoub, Ahmed E. Taha, Nahla Merghany, Nourhan M. Soliman, Haidy Elshebawy, Samar E. S. Abdelal, Lobna El-Ghoneimy, Aussan Al-Athwari, Nirmeen A. Kishk, Mona A. F. Nada, Marwa Farghaly, Amr Hassan, Mohamed I. Hegazy, Ahmed Abdelalim, Husam S. Mourad, Amira Hassouna, Alshimaa S. Othman, Tissa Wijeratne

**Affiliations:** 1 Department of Neurology, Cairo University Hospitals, Cairo 11511, Egypt; Doaamekkawy411@gmail.com (D.A.M.); sherifhamdy56@yahoo.com (S.H.); magednaseer@gmail.com (M.A.-N.); samirhatem@hotmail.com (H.S.S.); nevinmohy@gmail.com (N.M.S.); Alaa_elmazny@kasralainy.edu.eg (A.E.); sandra.ahmed@kasralainy.edu.eg (S.M.A.); nahlamerghany2@gmail.com (N.M.); nourhan.mohamed@residents.kasralainy.edu.eg (N.M.S.); haidyshebawy@gmail.com (H.E.); sayabd_1963@yahoo.com (S.E.S.A.); Lobna85@hotmail.com (L.E.-G.); nirmeenkishk@kasralainy.edu.eg (N.A.K.); mona_a_nada@yahoo.com (M.A.F.N.); marwafarghali@hotmail.com (M.F.); amrhasanneuro@kasralainy.edu.eg (A.H.); mhegazy@kasralainy.edu.eg (M.I.H.); a.aalim@kasralainy.edu.eg (A.A.); Husamneuro@kasralainy.edu.eg (H.S.M.); alshimaa.s.othman@kasralainy.edu.eg (A.S.O.); 2 Department of Chest Diseases, Cairo University Hospitals, Cairo 11511, Egypt; ahalfawy@kasralainy.edu.eg (A.A.H.); Jumanachest@outlook.sa (J.H.I.); Dr.amlibraheim@gmail.com (A.I.); Hodam.mabdelhamid@yahoo.com (H.M.A.-H.); rehab.m.hassan@kasralainy.edu.eg (R.M.); 3 Department of Neurology, Assuit University Hospitals, Assuit 71515, Egypt; anwarmoha2006@yahoo.com; 4 Endemic Medicine Department, Cairo University Hospitals, Cairo 11511, Egypt; Younankabara87@gmail.com (Y.K.A.); a.tahaa85@gmail.com (A.E.T.); 5 Department of Neurology, Aden University, Aden 37970, Yemen; Alathwariaussan2@gmail.com; 6 Faculty of Health and Environmental Sciences, School of Public Health and Interdisciplinary Studies, Auckland University of Technology, Auckland 1142, New Zealand; amira.hassouna@aut.ac.nz; 7 Department of Medicine and Neurology, AIMSS, Melbourne Medical School, Sunshine Hospital, Western Health, St Albans, VIC 3021, Australia; 8 Department of Public Health, La Trobe University, Bundoora, VIC 3086, Australia; 9 Department of Medicine and Dean’s Office, Faculty of Medicine, University of Rajarata, Saliyapura, Anuradhapuraya 50000, Sri Lanka

**Keywords:** COVID-19, neurological disorders, serial systemic immune inflammatory indices (SSIIi), stroke

## Abstract

Background: The COVID-19 pandemic has reached over 276 million people globally with 5.3 million deaths as of 22nd December 2021. COVID-19-associated acute and long-term neurological manifestations are well recognized. The exact profile and the timing of neurological events in relation to the onset of infection are worth exploring. The aim of the current body of work was to determine the frequency, pattern, and temporal profile of neurological manifestations in a cohort of Egyptian patients with confirmed COVID-19 infection. Methods: This was a prospective study conducted on 582 hospitalized COVID-19 patients within the first two weeks of the diagnosis of COVID-19 to detect any specific or non-specific neurological events. Results: The patients’ mean (SD) age was 46.74 (17.26) years, and 340 (58.42%) patients were females. The most commonly encountered COVID-19 symptoms were fever (90.72%), cough (82.99%), and fatigue (76.98%). Neurological events (NE) detected in 283 patients (48.63%) and were significantly associated with a severe COVID-19 at the onset (OR: 3.13; 95% CI: 2.18–4.51; *p* < 0.0001) and with a higher mortality (OR: 2.56; 95% CI: 1.48–5.46; *p* = 0.019). The most frequently reported NEs were headaches (*n* = 167) and myalgias (*n* = 126). Neurological syndromes included stroke (*n* = 14), encephalitis (*n* = 12), encephalopathy (*n* = 11), transverse myelitis (*n* = 6) and Guillain-Barré syndrome (*n* = 4). Conclusions: Neurological involvement is common (48.63%) in COVID-19 patients within the first two weeks of the illness. This includes neurological symptoms such as anosmia, headaches, as well as a constellation of neurological syndromes such as stroke, encephalitis, transverse myelitis, and Guillain-Barré syndrome. Severity of acute COVID-19 illness and older age are the main risk factors.

## 1. Introduction

The COVID-19 pandemic has spread across the world to more than 200 countries and territories in Asia, Europe, North America, Africa, and South America [1], culminating in 276 million infections with 5.3 million deaths and 248 million recovered as of 22 December 2021.

The cumulative prevalence of neurological disorders in COVID-19 has revealed that 36.4% of cases had neurological manifestations, including 24.8% involving the central nervous system (CNS) and 8.9% involving the peripheral nervous system (PNS) [2].

COVID-19 infection can present a broad spectrum of symptoms, ranging from asymptomatic, mild symptoms and severe symptoms requiring an intensive care unit (ICU) and ventilatory support. Common manifestations include fever, dry cough, dyspnea, diarrhea, muscle aches, anosmia, dysgeusia, ageusia, and fatigue, as well as characteristic laboratory and CT chest findings [2,3]. All components of the nervous system can be involved in COVID-19, as reported in the Wuhan observational study and in other case reports from different countries [4,5,6,7,8,9,10,11,12,13,14,15].

Pathobiology of COVID-19 predicts the neurological involvement to be expected rather than the exception [12,13,14,15,16]. COVID-19 and neurological involvement continue to occur from the acute illness to the long-term recovery with evidence of post-COVID-19Neurological Syndrome (PCNS) [13].

It is in this context we aimed to provide the first comprehensive report of acute neurological involvement in COVID-19 in a cohort of Egyptian patients in this research paper.

In Egypt, the first reported case of COVID-19 infection occurred on 14 February 2020. The patient was sent to a quarantine station. On 1st March 2020, the second COVID-19 case was announced. The first case of an Egyptian citizen to die from COVID-19 was reported on 20 March 2020, 20 days following the first mortality reported in Egypt (a German national). All schools and universities have been closed since 7 March 2020, and all mosques since 21 March 2020. To alleviate the impact of the epidemic and in a trial to decrease the spread of the virus, the Egyptian government has imposed different forms of lockdown [17].

The aim of this study was to determine the frequency and pattern of neurological affections in patients with proven COVID-19 infection in Egypt. In addition, we aimed to explore the temporal relationship between COVID-19 onset and the development of neurological deficits.

## 2. Material and Methods

### 2.1. Study Design and Participants

This was a hospital-based, prospective, observational study (with similar methodology to Garcia-Monco et al.) [18] conducted on 582 confirmed COVID-19 patients, according to the World Health Organization (WHO)’s Interim Guidance, second edition (World Health Organization, 2020), who were hospitalized between 25 March and 31 May 2020. Patients were recruited from five centers allocated by the Egyptian health care authorities for treatment of COVID-19 (Ministry of Health and Population, Egypt, 2020).

### 2.2. Patients’ Ascertainment and Enrollment

COVID-19 confirmed cases were initially identified with positive real-time reverse transcription polymerase chain reaction (RT-PCR) tests for SARS-CoV-2 by means of naso/oropharyngeal swabs.

Patients were then assessed by four pulmonologists (A.A., H.A., J.I., A.I.) and categorized according to the severity of their pulmonary symptoms into: mild, moderate, or severe infection (as per the Egyptian Ministry of Health and Population (MOHAP) Management Guideline) (Ministry of Health and Population, Egypt, 2020) (https://eu.docs.wps.com/l/sAHCZLEbvv6mNAYmkjZOrp, accessed on 28 December 2021). Initial laboratory tests included complete blood cell count (CBC), erythrocyte sedimentation rate (ESR), C-reactive protein (CRP), serum ferritin, D-dimer, creatine phosphokinase (CPK), lactate dehydrogenase (LDH), and liver and renal panel. Patients were meticulously followed up for the development of neurological manifestations during their hospital stay and for a minimum of two weeks following discharge. Brain CT or MRI scans were performed whenever indicated by clinical care needs.

#### Exclusion Criteria

Critically ill patients (defined as exhibiting more than one of the following symptoms: respiratory failure, septic shock, and/or multiple organ failure) were identified during initial triage evaluation in the quarantine hospitals or in pre-hospital settings. Thus, patients with comorbid illness and severe COVID-19 involvement were excluded from the beginning.

The protocol governing the study was executed in accordance with the 2013 Declaration of Helsinki. Our institutional review board (Ethical Committee of Neurology Department Cairo University Review Board) approved this study. Signed, written, and informed consent was obtained from all participants/informants prior to enrollment.

### 2.3. Data Collection

A standardized, predesigned, interviewer-administered checklist was developed following a review of the literature for use by the on-site attending physicians (A.T., H.A., Y.A., J.I., A.I.) as a screening tool for any neurological events (NE). The checklist comprised four sections: (a) demographic data and contact information; (b) constitutional symptoms, degree of evolution, clinical severity, and extent of COVID-19 infection; (c) medical inquiries related to any comorbid diseases; and (d) neurological manifestations, if any (including pattern, timing in relation to COVID-19 onset, and severity). Individuals who were found to experience NE were further assessed by five trained neurologists (R.M., D.A., N.S., S.E., S.O.) through focused patients’ examinations, and were followed up for at least 14 days following the onset of neurological manifestations. Prior to the final analysis, the clinical, laboratory, and radiological data of patients with NE were rigorously reviewed by four senior neurologists (M.A., H.S., N.S., G.S.) virtually, using one of the available telemedicine platforms.

### 2.4. Case Definition of Neurological Manifestations

Neurologic events (NE) are common in patients with COVID-19. Five major categories of neurological syndromes (1. Encephalopathies, 2. Inflammatory CNS syndromes, 3. Ischaemic strokes, 4. Peripheral neurological disorders such as acute inflammatory demyelinating neuropathy, brachial plexopathy, 5. Miscellaneous central nervous system disorders who did not fit in to previous categories) were described by Patterson et al. during the early stages of the pandemic in addition to the commonly associated neurological symptoms [19].

NE were defined as any newly developed neurological symptoms or reappearance/significant worsening of old symptoms (e.g., migraine, diabetic neuropathy, etc.) coinciding with contracting SARS-CoV-2 infection, and the diagnosed NE were classified as neurological symptoms or neurological syndromes as described by Patterson et al. [19]. Neurological symptoms occurred in association with fever and active systemic infection, without evidence of neurological syndromes such as stroke meningitis or meningoencephalitis, and these included headaches, myalgias, dizziness, and action tremor. Neurological syndromes were attributed to either CNS or PNS involvements as per Paterson et al. [19].

Suspected cerebrovascular diseases, including ischemic/hemorrhagic or cerebral venous sinus thrombosis (CVST), were diagnosed by head CT and MRI (and MRV when indicated) scans. Encephalitis was recognized by characteristic MRI features and/or cerebrospinal (CSF) analysis; encephalopathy was defined by changes in the conscious content with confusion and/or delirium in the absence of structural brain lesions or infection. Muscle injury was identified by skeletal muscle pain and elevated serum CPK levels (>200 U/L) [4]. Other neurological manifestations were diagnosed by their specific symptoms and signs, as well as by appropriate investigations.

Based on the explicit temporal relationship between COVID-19 onset and the development of neurological deficits, the NE were further classified into: (a) presenting symptoms of COVID-19; (b) early manifestations (within the first seven days of onset of infection); and (c) late manifestations (within 8–14 days).

### 2.5. Statistical Analysis

Data were collected, coded, and analyzed using the IBM Statistical Package for the Social Sciences (SPSS) software version 21.0 for Windows (IBM, Armonk, NY, USA).

The Shapiro–Wilk test was used to determine the normality of the distributions. Simple descriptive analysis of baseline demographic, clinical, and laboratory/radiographic data were summarized as frequencies (%) and means ± standard deviations (SD) for normally distributed variables or medians (with interquartile ranges (IQR)) for non-normally distributed parameters. The Student’s *t*-test was used to compare continuous variables and the chi-square test (*χ*^2^) to compare categorical variables.

The strength of association between risk factors/exposures and outcomes (e.g., the association between comorbid conditions and severity of illness, severity of illness and NE, and the presence of NE and mortality) was presented as an odds ratios (OR) with 95% confidence intervals (CI).

Analysis of variance (ANOVA) was used to determine the differences in the clinical data among the different degrees of disease severity. A value of *p* < 0.05 was considered to be statistically significant.

## 3. Results

### 3.1. Patients’ Characteristics

The mean (±SD) age of the COVID-19 patients enrolled in the study was 46.74 (±17.26) years; 340 (58.42%) of the patients were females, 255 (43.81%) had one or more underlying chronic medical conditions, and 18.21% were current smokers. Contact with confirmed COVID-19 cases was reported in about half of the enrolled patients, and familial clustering in about one quarter. A summary of the patients’ basic clinical characteristics is given in Table 1.

### 3.2. Clinical Manifestations and Disease Course

The mean (±SD) time from COVID-19 symptom onset to first attendance with a physician was 2.08 (± 0.91) days.

The most commonly encountered symptoms were fever (*n* = 528, 90.72%), cough (*n* = 483, 82.99%), and fatigue (*n* = 448, 76.98%). Patients with severe illness were significantly older (*p* < 0.0001), males (*p* < 0.002), with more comorbid conditions (OR: 1.81; 95% CI: 1.28–2.57; *p* < 0.001), and were more likely to have neurological manifestations compared with patients with non-severe forms (OR: 3.13; 95% CI: 2.18–4.51; *p* < 0.0001) (Table 2).

At the two-week follow-up stage, 441 (75.77%) patients were discharged (stable and fulfilling discharge criteria) (Ministry of Health and Population, Egypt, 2020) with a mean (±SD) hospital stay of 10.88 (± 2.57) days, 107 (18.38%) required ICU admission, 84 (14.43%) received invasive mechanical ventilation, and 33 (5.67%) patients died. One or more “non-neurological” complications were reported in 146 (25.09%) patients that called for more intensive/close monitoring, including secondary bacterial infections (*n* = 73), acute respiratory distress syndrome (ARDS) (*n* = 36), elevated liver enzymes (*n* = 36), renal impairment (*n* = 32), dysrhythmias (*n* = 23), and septicemia (*n* = 17).

### 3.3. Neurological Events (NE)

During their hospital stay and follow-up periods, 283 patients (48.63%) experienced NE, with a mean (±SD) age of 49.04 (± 16.83) years. Two or more NE were detected in 106 patients (18.21%).

The frequency of neurologic involvement was comparable between patients with and without different comorbid conditions (*p* > 0.5); yet, they were significantly associated with more severe COVID-19 illness (OR: 3.13; 95% CI: 2.18–4.51; *p* < 0.0001) and with a higher mortality (OR: 2.56; 95% CI: 1.48–5.46; *p* = 0.019).

#### 3.3.1. Neurological Symptoms (*n* = 187)

The most frequently reported neurological symptoms were headaches (*n* = 167, 28.69%) and body pains “myalgias without elevated muscle enzyme” (*n* = 126, 21.65%). Headache was moderate to severe, with diffuse aching in 74.85% of patients (125/167) and pulsating in 22.75% (38/167). Sometimes it even predated the onset of typical constitutional symptoms. Other neurological symptoms were dizziness and action tremors. Neurological symptoms were generally mild, transient, and tended to be prominent early in the disease course, alongside other constitutional symptoms (88.23% occurred within the first week), with rapid regression in parallel with improvement/resolution of the infection during the first week. However, dizziness and action tremors showed a relatively prolonged course.

#### 3.3.2. Neurological Syndromes

These were further divided into neurological syndromes with acute presentations and late appearance of neurological syndromes during the disease course.

#### 3.3.3. Acute Presentations

Neurological syndromes at the onset of illness were displayed in 18 patients (3.09%) (unusual presentations of COVID-19); six patients (1.03%) presented with strokes (large vessel occlusion in five patients and hemorrhage in one patient); five patients (0.86%) presented with encephalitis (according to clinical presentation and characteristic brain imaging features and/or CSF lymphocytic pleocytosis), three patients (0.52%) experienced ascending areflexic tetraparesis (Guillain–Barré syndrome), two patients (0.34%) had encephalopathy, and two patients (0.34%) presented with transverse myelitis.

#### 3.3.4. Neurological Syndromes Developed during the Two-Week Follow-Up Period

Fourteen patients, without evident vascular risk factors, had cerebrovascular strokes (nine were ischemic, two were hemorrhagic, and three had CVST; their age range (mean ± SD) was 31–39 (34.33 ± 2.58) years), of whom eight died. Viral encephalitis was diagnosed in 12 patients (2.07%); all of them had altered mental status and behavioral changes, and five patients experienced seizures. Diagnosis was based on characteristic brain imaging features in 10 patients, and three had CSF lymphocytic pleocytosis. Despite all of these patients receiving Acyclovir, three of these patients died. Eleven patients showed encephalopathic manifestations along with prominent agitation that necessitated sedation and small doses of antipsychotics (1–2 mg haloperidol IV). Six patients (1.03%) had transverse myelitis with subacute, asymmetric lower extremity weakness with clearly defined sensory level and sphincter dysfunction (spinal MRI showed dorsal long segment T2 hyperintensity with patchy enhancement in all but one patient, who had normal spinal MRI but with typical presentation). Other detected specific NE were Guillain–Barré Syndrome (*n* = 4). Neurological examination disclosed symmetric weakness and areflexia in both legs and feet. Several hours later, they developed sensation to light touch and pinprick that was decreased distally. Their condition progressed in both the lower and upper limbs. Nerve conduction studies showed delayed distal latencies and absent F waves in the early course, supporting demyelinating neuropathy. None of them required intubation; they were treated with intravenous immunoglobulin and showed a favorable response. Muscle injury with elevated CPK was detected in eight patients, and acute sensory neuropathy was diagnosed in nine patients. The neurological manifestations in temporal relationship with contracting COVID-19 infection are given in Table 3.

### 3.4. Laboratory Data

The most notable initial laboratory abnormalities were elevated erythrocyte sedimentation rate (ESR) (*n* = 530) (91.07%, (95% CI 88.54–93.21%)), increased CRP (*n* = 413) (70.96%, (95% CI 67.24–74.61%)), lymphopenia (*n* = 369) (63.40%, (95% CI 59.32–67.41%)), and high serum ferritin (*n* = 315) (54.12%, (95% CI 50.04–58.38%)), followed by increased D-dimer levels (*n* = 281) (48.28%, (95% CI 44.57–52.81%)).

Severe illness was associated with significantly higher median IQR levels of CRP than non-severe illness (83.91 mg/L (32.56–158.93) vs. 29.45 mg/L (23.50–121.56); *p* < 0.001); higher serum ferritin (575 ng/mL (350–775) vs. 350 ng/mL (180–590); *p* < 0.01), and lower lymphocytic count (0.69 × 10^9^/L (0.46–0.83) vs. 0.97 × 10^9^/L (0.75–1.19); *p* < 0.01). There were significantly higher D-dimer levels in patients with cerebrovascular disorders (*n* = 14) than in those with other central NE (*n* = 29) (mean (±SD) 0.98 (±0.40) mg/L vs. 0.49 (±0.31) mg/L; *p* < 0.001); other laboratory results were comparable (*p* > 0.05).

### 3.5. Radiological Data

High-resolution chest CT (HRCT) scans were carried out on 456 patients (78.35%). Normal scans were detected in 97 patients (16.67%), whereas lobular ground glass opacities (GGO) were found in the remainder (*n* = 359). Dense consolidation with interlobular septal and vascular thickening (“crazy paving signs”) was detected in 84 patients (14.43%). No statistically significant differences were found between patients with and without NE in respect of the radiological findings (*p* > 0.05).

## 4. Discussion

This paper reported the different neurological manifestations among 582 hospitalized COVID-19 patients with variable disease severity. In our study, we followed up enrolled patients over a two-period to detect any new NE; if patients reported any neurological manifestations, they were followed up for a further 14 days from the onset of their NE. The first Chinese study to report neurological involvement in COVID-19 patients was a retrospective one. Later, a multicenter retrospective-prospective Italian study was designed in order to follow up patients for six months [20]. SARS-CoV-2 appears to cause a variety of both CNS and PNS disorders, including cerebrovascular stroke, encephalitis, Guillain–Barré Syndrome, and isolated cranial and nerve palsy [21].

According to the extent of chest involvement, 188 (32.3%) patients experienced mild COVID-19 infection, 203 (34.9%) experienced moderate infection, while 191 (32.8%) suffered from its severe form, according to the MOHAP Management Guide (Ministry of Health and Population, Egypt, 2020) (Table 1, https://eu.docs.wps.com/l/sAHCZLEbvv6mNAYmkjZOrp (accessed on 28th December 2021) for severity criteria as per MOHAP). Regarding neurological affection in COVID-19 patients, 283 patients (48.63%) experienced neurological manifestations and 106 (18.21%) experienced two or more NE.

Neurological manifestations were more prominent in patients with severe COVID-19 as well as in older patients, which can be attributed to the presence of high viral loads in already immunologically exhausted victims. That could return to explanations of patients as recurrent dealing with infected persons. In accordance with this finding, many studies have found that neurological manifestations were more obvious in elderly patients and those with severe disease forms [3,4,6,22,23]. The shared pathobiology between stroke, COVID-19, and brain involvement is a case in point to explain this further [12]. A stroke occurred in the context of compromised vascular system such as elderly [12,24,25].

We did not find a correlation between any comorbidity and the occurrence of neurological manifestations in COVID-19 cases, though an association between concurrent comorbidities and the development of a severe form of COVID-19 did exist. This observation may reflect the magnitude of such a virulent virus that is capable of harming the nervous system in the absence of a previous significant medical condition. Additionally, we excluded patients who had comorbid illness with severe COVID-19 involvement from the beginning. However, other studies have emphasized an increased likelihood of neurological manifestations in patients with comorbidities [3,12,16,26].

In the present study, headache was found to be the most prominent neurological symptom, either as a presenting symptom or as an early manifestation of COVID-19, followed by myalgia, dizziness, and then action tremors. Headache occurrence during the early stages of COVID-19 infection could be attributed to excess pro-inflammatory cytokine release, e.g., interleukin 1 (IL 1) and tumor necrosis factor-alpha (TNF-α), which promote trigeminal neural network sensitization, a key event in headache pathology [27]. Concerning infection-associated myalgia, IL 1 is the incriminated agent that detaches amino acids from muscle, leading to muscle aches [28]. Shapiro et al. observed that a higher prevalence of headache disorders were associated with a higher COVID-19 mortality rate (37.8%; F value = 16.68%) [29]. By contrast, the authors observed a negative trend between the prevalence of headache disorders and influenza death rates [29]. Interestingly, when considering headache as a symptom of COVID-19, in the 48 meta-analyzed studies, Shapiro et al. observed a significantly higher risk ratio of survival (RR:2.178 (1.8882–2.520), *p* < 0.0001) among COVID-19 in patients with headaches [29].

We reported dizziness in only 20% of our patients, whereas it was a more prominent feature in other studies [4,30,31,32,33,34,35,36,37,38,39,40,41,42,43]. However, the vague and imprecise feeling of dizziness was easily disregarded by patients who were overwhelmed by the much more prominent symptoms of their COVID-19 medical condition. Action tremors in our cohort had a relatively prolonged course and may have resulted from the anxiety and stress associated with COVID-19. This observation was in agreement with the findings of previous studies, as a presumed direct viral invasion of the cerebellum [44].

Neurological manifestations in COVID-19 patients can be mediated via multiple mechanisms, including viral neurotropism, cerebral hypoxia provoked by respiratory failure, or an immune-mediated mechanism through a cytokine storm, acting via angiotensin-converting enzyme 2 (ACE-2) receptors with subsequent endothelial dysfunction [8,9,12,16].

SARS-CoV-2 was found to cause CNS symptoms, including cerebrovascular strokes (ischemic, hemorrhagic, and CVST). COVID-19 has been associated with the broad family of cerebrovascular diseases [6,7,8,45,46,47] In our study, all three stroke subtypes were detected with a greater preponderance of ischemic types, which agrees with the findings of previous studies [3,12]. However, acute hemorrhagic necrotizing encephalopathy with scattered hemorrhages has previously been reported [48]. The occurrence of ischemic stroke among COVID-19 patients could be attributed to the cytokine storm that causes endothelial dysfunction resulting from direct invasion of endothelial cells by the virus, which results from the interaction between the coronavirus S protein and the ACE-2 receptors, expressed in the capillary endothelium. Endothelial dysfunction increases thrombin synthesis and decreases fibrinolysis, which leads to a hypercoagulable state. This leads to the high rate of thrombotic complications observed in patients with COVID-19 [12,16].

The pathophysiological mechanism of stroke in COVID-19 patients is thought to be mediated through alteration of ACE-2 receptors, with subsequent thrombosis and/or hemorrhage. Nevertheless, thrombocytopenia, cytokine storm, and hypercoagulable status are other postulated mechanisms [12,16].

In our study, 11 patients developed an encephalopathic state, for which many underlying mechanisms were postulated, including hypoxia and cerebral edema that was found in the autopsies of encephalopathic victims. In addition, toxic or metabolic causes may also play a role [49].

We reported 12 cases of encephalitis, which was previously recorded in two case reports [48,50]. The proposed hypothesis suggested direct brain infiltration [51,52], an assumption that can also be applied to our reported cases of transverse myelitis where the spinal cord could also have undergone viral infiltration.

Regarding PNS symptoms, anosmia and ageusia were prominent symptoms, either as presenting or as early manifestations, with only a few cases experiencing these symptoms as late manifestations. They were commonly reported in COVID-19 cases even in the absence of other symptoms, which was attributed to direct viral invasion of olfactory receptors, which then gained access to the rhino-cortex through neuronal transport [53].

In the current study, symmetrical lower-limb distal sensory neuropathy and muscle injury (myositis-like presentations with elevated CPK) were found to be both early and late COVID-19 manifestations. This finding is in agreement with that of Mao and coworkers [4].

## 5. Conclusions

COVID-19 and neurological involvement is the expectation rather than the exception. We demonstrated undeniable evidence of acute neurological involvement with severe disease in vulnerable populations during the early phase of the pandemic. It is worth reflecting that neurological disorders have been the leading cause of disability well before the pandemic hit our world. The pandemic exacerbates existing conditions for the patients, care givers, and family members who are already facing the enormity of challenges with neurological disability worldwide.

A rapid deterioration in the health of a COVID-19 patient should draw attention in order to exclude recent neurological insults such as stroke or encephalitis. COVID-19 could be considered as one of the differential diagnoses of any case presented with neurological affection during the current pandemic crisis.

The global academic neurology community must continue to collaborate on a cross-culturally validated surveillance tool kit to monitor and explore the acute and long-term neurological manifestations as a matter of priority [10,15].

## Figures and Tables

**Table 1:** Demographics and clinical characteristics of the study population.

Variable		
Age (range, mean, SD ^†^) years		19–83, 46.74 (17.26)
Gender (*n*, %)	Male	242 (41.58%)
	Female	340 (58.42%)
BMI ^‡^ (mean, SD ^†^)		27.81(3.51)
Comorbidities (*n*, %)	Any	255 (43.81%)
	Hypertension	145 (24.91%)
	Diabetes	130 (22.34%)
	Smoking	106 (18.21%)
	Cardiovascular diseases	28 (4.81%)
	Chronic obstructive pulmonary disease	24 (4.12%)
	Chronic liver diseases	15 (2.58%)
	Chronic renal diseases	11 (1.89%)
	Autoimmune diseases	19 (3.26%)
History of contact with confirmed COVID-19 patients (*n*, %)		293 (50.34%)
Familial clustering (*n*, %)		143 (24.57%)

^†^ Data expressed as number and % or means ± SD according to need; SD: Standard Deviation; ^‡^ BMI: Body Mass Index.

**Table 2:** Clinical manifestations of different degrees of COVID-19 infection.

Symptoms		Mild (*n* = 188)	Moderate (*n* = 203)	Severe (*n* = 191)	Total (*n* = 582)	*p* Value
Age (mean, SD ^†^)		43.17 (16.81)	44.12 (16.07)	53.04 (17.26)	46.74 (17.26)	0.0001 ***
Sex	Female	128 (68.09%)	115 (56.65%)	97 (50.79%)	340 (58.42%)	0.002 **
	Male	60 (31.91%)	88 (43.35%)	94 (49.21%)	242 (41.58%)	
Comorbidities (any)		71 (37.77%)	81 (39.90%)	103 (53.93%)	255 (43.81%)	0.002 **
Fever		169 (89.89%)	181 (89.16%)	178 (93.19%)	528 (90.72%)	0.345
Cough		152 (80.85%)	171 (84.24%)	160 (83.77%)	483 (82.99%)	0.633
Fatigue		142 (75.53%)	151 (74.38%)	155 (81.15%)	448 (76.98%)	0.238
Sore throat (throat pain)		70 (37.23%)	77 (37.93%)	71 (37.17%)	218 (37.46%)	0.985
GIT ^‡^ manifestations/Diarrhea		57 (30.32%)	55 (27.09%)	60 (31.41%)	172 (29.55%)	0.619
Headache		43 (22.87%)	56 (27.59%)	68 (35.60%)	167 (28.69%)	0.269
Chest tightness/dyspnea		44 (23.40%)	42 (20.69%)	43 (22.51%)	129 (22.16%)	0.804
Body pains (myalgias)		34 (18.09%)	53 (26.11%)	39 (20.42%)	126 (21.65%)	0.228
Rhinorrhea		34 (18.09%)	37 (18.23%)	24 (12.57%)	95 (16.32%)	0.23
Neurological		68 (36.17%)	82 (40.39%)	133 (69.63%)	283 (48.63%)	0.001 **

^†^ SD: Standard Deviation; ^‡^ GIT: Gastrointestinal tract; **, ***: Statistically significant.

**Table 3:** Neurological manifestations in temporal relation to COVID-19 infection.

Neurological Manifestation	Presenting Features	Early (1–7 Days)	Late (8–15 Days)	Total
**Neurological Symptoms ^†^ (*n* = 187, 32.13%)**				
Any	103 (17.70%)	62 (10.65%)	22 (3.78%)	187 (32.13%)
Headache associated with fever (9.2.2.1: acute headache attributed to systemic viral infection *)	91 (15.64%)	57 (9.79%)	19 (3.26%)	167 (28.69%)
Myalgias	64 (10.99%)	44 (7.56%)	18 (3.09%)	126 (21.65%)
Dizziness	47 (8.086%)	56 (9.62%)	13 (2.23%)	117 (20.10%)
Action tremor	31 (5.32%)	44 (7.56%)	10 (1.72%)	85 (14.60%)
**Neurological Syndromes ^†^ (*n* = 193, 33.16%)**				
**Central nervous system manifestations (*n* = 43, 7.39%)**				
Stroke (ischemic)	5 (0.86%)	3 (0.52%)	1 (0.17%)	9 (1.55%)
Stroke (hemorrhagic)	1 (0.17%)	1 (0.17%)	0 (0%)	2 (0.34%)
CVST ^‡^	0 (0%)	0 (0%)	3 (0.52%)	3 (0.52%)
Encephalitis	5 (0.86%)	4 (0.69%)	3 (0.52%)	12 (2.07%)
Encephalopathy/delirium (prominent agitation and confusion)	2 (0.34%)	7 (1.20%)	2 (0.34%)	11 (1.88%)
Transverse myelitis	2 (0.34%)	1 (0.17%)	3 (0.52%)	6 (1.03%)
**Peripheral nervous system manifestations (*n* = 169, 29.04%)**				
Guillain-Barré syndrome	3 (0.52%)	0 (0%)	1 (0.17%)	4 (0.69%)
Muscle injury (↑ CPK ^§^)	0 (0%)	6 (1.03%)	2 (0.34%)	8 (1.37%)
Symmetrical lower limbs distal sensory neuropathy	0 (0%)	4 (0.69%)	5 (0.86%)	9 (1.55%)
Anosmia/ageusia	92 (15.81%)	56 (9.62%)	0 (0%)	148 (25.43%)
All (*n* = 283)				

^†^ NE: Neurological Events, ^‡^ CVST: Cerebral Venous Sinus Thrombosis; ↑ CPK ^§^: Elevated Creatine PhosphoKinase; *: according to International Headache Society Classification (IHS 2018), 2018.

## Data Availability

Data supporting reported results can be accessible with a reasonable request from the corresponding author from Egypt.
